# Loop-mediated isothermal amplification (LAMP) assays targeting 18S ribosomal RNA genes for identifying *P. vivax* and *P. ovale* species and mitochondrial DNA for detecting the genus *Plasmodium*

**DOI:** 10.1186/s13071-021-04764-9

**Published:** 2021-05-24

**Authors:** Xi Chen, Jiaqi Zhang, Maohua Pan, Yucheng Qin, Hui Zhao, Pien Qin, Qi Yang, Xinxin Li, Weilin Zeng, Zheng Xiang, Mengxi Duan, Xiaosong Li, Xun Wang, Dominique Mazier, Yanmei Zhang, Wei Zhao, Benjamin M. Rosenthal, Yaming Huang, Zhaoqing Yang

**Affiliations:** 1 Laboratory of Pathogen Biology and Immunology, Kunming Medical University, Kunming, 650500 Yunnan People’s Republic of China; 2 Department of Pathogen Biology and Immunology, Kunming Medical University, Kunming, 650500 Yunnan People’s Republic of China; 3 Zhejiang Provincial Center for Disease Control and Prevention, No. 3399 BinSheng Road, Binjiang District, Hangzhou, 310051 Zhejiang People’s Republic of China; 4 Shanglin County People’s Hospital, Shanglin, 530500 Guangxi People’s Republic of China; 5 INSERM, CNRS, Centre d’Immunologie et des Maladies Infectieuses (CIMI), Sorbonne Université, 75013 Paris, France; 6 Animal Parasitic Disease Laboratory, USDA-Agricultural Research Service, 10300 Baltimore Avenue, Beltsville, MD 20705 USA; 7 Guangxi Zhuang Autonomous Region Center for Disease Prevention and Control, Nanning, 530021 Guangxi People’s Republic of China

**Keywords:** LAMP, Genus *Plasmodium* spp., *Plasmodium vivax*, *Plasmodium ovale*

## Abstract

**Background:**

Loop-mediated isothermal amplification (LAMP) has been widely used to diagnose various infectious diseases. Malaria is a globally distributed infectious disease attributed to parasites in the genus *Plasmodium*. It is known that persons infected with *Plasmodium vivax* and *P. ovale* are prone to clinical relapse of symptomatic blood-stage infections. LAMP has not previously been specifically evaluated for its diagnostic performance in detecting *P. ovale* in an epidemiological study, and no commercial LAMP or rapid diagnostic test (RDT) kits are available for specifically diagnosing infections with *P. ovale*.

**Methods:**

An assay was designed to target a portion of mitochondrial DNA (*mtDNA*) among *Plasmodium* spp., the five human *Plasmodium* species and two other assays were designed to target the nuclear 18S ribosomal DNA gene (*18S rDNA*) of either *P. vivax* or *P. ovale* for differentiating the two species. The sensitivity of the assays was compared to that of nested PCR using defined concentrations of plasmids containing the target sequences and using limiting dilutions prepared from clinical isolates derived from Chinese workers who had become infected in Africa or near the Chinese border with Myanmar.

**Results:**

The results showed that 10^2^ copies of the mitochondrial target or 10^2^ and 10^3^ copies of *18S rDNA* could be detected from *Plasmodium* spp.,* P. vivax* and *P. ovale*, respectively. In 279 clinical samples, the malaria Pan *mtDNA* LAMP test performed well when compared with a nested PCR assay (95% confidence interval [CI] sensitivity 98.48–100%; specificity 90.75–100%). When diagnosing clinical cases of infection with *P. vivax*, the *18S rDNA* assay demonstrated an even great sensitivity (95.85–100%) and specificity (98.1–100%). The same was true for clinical infections with *P. ovale* (sensitivity 90.76–99.96%; specificity 98.34–100%). Using plasmid-positive controls, the limits of detection of Malaria Pan, *18S rDNA P. vivax* and *18S rDNA P. ovale* LAMP were 100-, 100- and tenfold lower than those of PCR, respectively.

**Conclusion:**

The novel LAMP assays can greatly aid the rapid, reliable and highly sensitive diagnosis of infections of *Plasmodium* spp. transmitted among people*,* including *P. vivax* and *P. ovale*, cases of which are most prone to clinical relapse.

**Graphic abstract:**

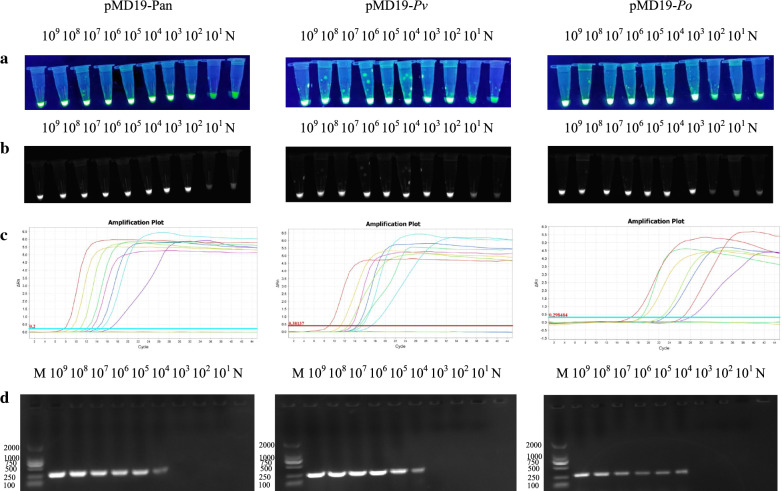

**Supplementary Information:**

The online version contains supplementary material available at 10.1186/s13071-021-04764-9.

## Background

Malaria, transmitted by female *Anopheles* mosquitoes, is a major global burden to human health and welfare, with an estimated 228 million cases and 405 thousand deaths reported in 2018 [1]. Anopheline vectors transmit, from person to person, malaria derived from any of four parasite species in the genus *Plasmodium*: *Plasmodium falciparum*, *P. vivax*, *P. malariae* and *P. ovale* (including *P. ovale curtisi* and *P. ovale wallikeri*). Of these, *P. vivax* is the most widespread and responsible for the majority of malaria infections outside of Africa, with 5.9 million clinical cases reported in 2018 [1]. *Plasmodium vivax* is endemic in Southeast Asia, where it is generally assumed to be the causative agent of malaria [2, 3]. *Plasmodium ovale* is mainly restricted to West Africa, where it generally has a low prevalence [4]. Among the *Plasmodium* species of human malaria, these latter two are distinctive by sharing a dormant hypnozoite stage in hepatocytes that can cause relapse of symptomatic blood-stage infections weeks, months or even years after the primary infection [5, 6]. The administration of primaquine can eliminate hypnozoites in the liver [7]. It is therefore important for clinicians to identify whether either of these two species is present, so as to promptly initiate the proper therapeutic regimen.

The prompt and accurate diagnosis of malaria constitutes a major technical challenge [8]. Delayed diagnosis and therapy can exacerbate the severity of the disease. Misdiagnosis of the *Plasmodium* species results in improper use of anti-malarial drugs, worsening the spread of drug resistance. Furthermore, mixed infections comprised of more than one species of parasite are often not recognized, thereby preventing accurate diagnosis and treatment [9].

Traditional malaria diagnosis employs microscopic examination of thin and thick blood smears. Although microscopy can be used to diagnose the density and developmental stages of each parasite species, it is an arduous procedure and demands high technical proficiency. False negative diagnoses, most common when parasitemia is low [10] (for example after treatment), impedes proper case management.

Other diagnostic techniques employ antigen capture. The main targets for antigen capture methods include the *P. falciparum*-specific histidine-rich protein-2 (*PfHRP2*), *P. falciparum*-specific lactate dehydrogenase (*Pf-pLDH*), *P. vivax*-specific lactate dehydrogenase (*PvpLDH*), pan-*pLDH* and pan-aldolase. The sensitivity and specificity of these assays are poor, especially for mixed infections, and existing methods do not diagnose infections with *P. ovale*, *P. malariae* or *P. knowlesi* [11].

A third family of methods employs PCR assays to identify nucleic acids of the parasite. PCR assays have a higher sensitivity and specificity than antigen capture methods, detecting as few as 1–5 protozoa/μl blood, and are able to diagnose mixed infections. However, they are time-consuming (typically requiring 3–4 h of run time on a thermocycler) and costly, and require sophisticated experimental testing equipment and professional technicians. Consequently, PCR assays are of limited utility in many regions endemic for malaria [12]. Therefore, it is of great importance to develop a simple method for the rapid and accurate detection of *Plasmodium* species.

Fluorescence in situ hybridization (FISH) has also proven effective in diagnosing human malaria [13]. This cytogenetic technique is based on the detection of DNA or RNA following hybridization with complementary sequences labeled with fluorescent probes and can used in combination with microscopy without the need for nucleic acid amplification. FISH has been documented as having a high sensitivity (98%) for detecting *Plasmodiun* infections, detecting live parasites, with a requirement for 55–65 protozoa/μl blood [14]. Microscopy skills are widely available in many malaria-endemic countries, but specialized skills are required for fluorescent microscopy, which may limit use of the FISH in certain endermic areas.

Loop-mediated isothermal amplification (LAMP), first described in 2000 [15], employs isothermal cyclic strand displacement DNA synthesis using *Bst* DNA polymerase. Such tests can be performed at 60–65 °C in 1 h, using a simple water bath. Unlike PCR, it does not require DNA templates to be denatured. It uses a series of six primers, of which four target their own templates and two loop primers accelerate production of the product. As amplification products accumulate, they can be visualized by eye as an increase in turbidity and fluorescence under illumination by ultraviolet (UV) light [16]. The LAMP technique is widely used in the diagnosis of various infectious diseases, such as tuberculosis, toxoplasmosis and trichomoniasis [17–19]. LAMP has been used in malaria diagnosis since 2007 [16].

The Eiken Loopamp™ MALARIA Pan Detection kit (Eiken Chemical Company, Tokyo, Japan) and the Illumigene Malaria Plus test (Meridian Bioscience Inc., Cincinnati, OH, USA) are the most widely-used commercial LAMP kits for detecting malaria. However, the Eiken Loopamp™ MALARIA Pan Detection kit was recently found to lack the reliability required for diagnostic laboratories, especially given limited sensitivity when parasitemia is low [20]. In addition, published LAMP primers intended to be specific for *P. ovale* [16] were reported to yield a high risk of cross reactions [20]. To date, a LAMP assay or rapid diagnostic test (RDT) specific for detecting *P. ovale* is not commercially available, nor has any report documented LAMP assay performance on clinical samples containing *P. ovale*.

In this study, we developed and tested novel LAMP assays to detect all species of all human malaria and to differentially diagnose *P. vivax* and *P. ovale* infections, using a positive control plasmid construct as well as clinical cases of malaria imported to China as a basis to evaluate sensitivity and specificity. We sought to establish, for resource-limited conditions, an accurate and rapid method to diagnose any human malaria infection and also identify when species prone to causing clinical relapse are present. We also tried to reduce the cost of the assay to make it widely available.

## Methods

### Collection of clinical samples and microscopic examination

All clinical samples for use in the study were collected from patients with suspected malaria with febrile symptoms. The clinical samples were obtained from the Department of Infectious Diseases in Shanglin Hospital, Shanglin County, Guangxi Province, China and Tengchong County (China–Myanmar border), Yunnan Province, China. The clinical subjects were Chinese migrant workers who had returned from Africa or Myanmar within the preceding 2 weeks. All clinical samples were diagnosed immediately by microscopic examination and sent to Kunming Medical University for molecular studies. Reference *Plasmodium falciparum* (strain 3D7) cultured* in vitro* and the Strain H clone of *Plasmodium knowlesi* were obtained from the Malaria Research and Reference Reagent Resource Center. *Toxoplasma gondii*, a protozoan infecting nucleated cells at a high infection rate in humans, obtained from the Department of Pathogen Biology and Immunology, Kunming Medical University, was also used for testing the novel primers (as a negative control). Thin and thick blood films were prepared and Giemsa-stained for microscopy examination. All slides were read by skilled microscopists; thin films were read under 10 × 100 magnification. Species were identified by observing characteristic morphology in thin films. The parasite density was calculated in thick films according to the numbers of parasites per 500 leukocytes, assuming 8000 leukocytes/μl blood.

### DNA extraction and nested PCR for identifying *Plasmodium* species

Genomic DNA was prepared using the Roche High Pure PCR Template Preparation kit (Roche Diagnostics, Indianapolis, IN, USA). In brief, 200 μl whole blood, 200 μl Binding Buffer and 40 μl proteinase K were heated at 70 °C for 10 min. Then, 100 μl of isopropanol, 500 μl of Inhibitor Removal Buffer and 500 μl of Washing Buffer were added sequentially. After each addition, the solution was centrifuged at 8000 rpm for 1 min. A 50-μl aliquot of elution buffer was used to elute the DNA, and the extracted DNA was stored at − 20 °C until use. Aliquots of 1 μl DNA were used to run the nested PCR for identifying the five human *Plasmodium* species according to previously reported methods [21, 22].

### LAMP primer design

LAMP Designer 1.15 was used to identify mtDNA targets conserved among all five species (Malaria Pan assay). According to the LAMP primer design requirement, the targeting reference sequences should be shorter than 300 bp. The *18S rDNA* gene sequences retrieved from *P. vivax* and *P. ovale* were used for designing primers for *P. vivax* and *P. ovale*. Reference sequences downloaded were *mtDNA* sequences from *P. falciparum* [Genbank no. M99416.1], *P. vivax* [Genbank no. KF668441.1], *P. ovale curtisi* [Genbank no. HQ712052.1], *P. ovale wallikeri* [Genbank no. HQ712053.1], *P. malariae* [Genbank no. AB489194.1] and *P. knowlesi* [Genbank no. AY722797.1] and *18S rDNA* sequences from *P. vivax* [Genbank no. DQ162604.1], *P. ovale curtisi* [Genbank no. JF894405.1] and *P. ovale wallikeri* [Genbank no. JF894406.1].

### Establishment of a closed-tube visualization LAMP system

A set of LAMP primers consisting of F3, B3, FIP, BIP, LF and LB primers was used (Table 1). The 12.5-μl reaction mixture consisted of 1.6 μM FIP and BIP, 0.8 μM LpF and LpB, 0.2 μM F3 and B3, 4 mM MgSO4, 1.25 μL buffer 10×, 0.5 μL *Bst* DNA polymerase (New England Biolabs, Ipswich, MA, USA), 1 μl template DNA, 0.4 μM Calcein-MnCl_2_ dye and 6 μl double-distilled water (DDW). The LAMP reaction systems were performed in capped PCR tubes in a water bath at 65 °C, for 60 min without further manipulation. After cooling, each tube was examined by eye under visible light to determine whether the contents had changed from clear and orange to green and turbid, and whether under UV light the solution had become fluorescent. For confirmation, five samples of each *Plasmodium* species were tested by LAMP primers and analyzed by 1.5% gel electrophoresis. Following electrophoresis, successful amplifications could be identified by the presence of continuous trapezoidal bands.

**Table 1:** Primer sets of the loop-mediated isothermal amplification for detection of Malaria Pan, *Plasmodium vivax* and *P. ovale*

Species	Primer	Sequence (5′–3′)
Malaria Pan LAMP assay	F3	TGTCAACTACCATGTTACGAC
	B3	AACGGTCCTAAGGTAGCAA
	FIP (F1c-F2)	TACGGCCCGACGGTAAGATCGTAACCATGCCAACAC
	BIP (B1c-B2)	AGGAGTCTCACACTAGCGACAAAATTCCTTGTCGGGTAATCTC
	LPF	CTGAGCACCTTAACTTCCCTAA
	LPB	TACACCGTTCATGCAGGAC
*P. vivax*	F3	CTAATTAGCGGTAAGTACGACA
	B3	AGCCTAGTTCATCTAAGGACA
	FIP (F1c-F2)	ACCAAACGCATCAGCTATTCGTATGTCGGATTGGATCTGGA
	BIP (B1c-B2)	TTACTTGGCTTATCGTACCGTTCAGACCTGTTGTTGCCTT
	LPF	CACCGACACGAAGTATAATTGC
	LPB	GCTTCTTAGAGGAACGATGTGT
*P. ovale*	F3	CGAGTTTCTGACCTATCAGC
	B3	GCTGGCACCAGACTTG
	FIP (F1c-F2)	GATGTGGTAGCTATTTCTCAGGCTCCCTAACATGGCTATGACGG
	BIP (B1c-B2)	GCAGCAGGCGCGTAAATTACAACCATGAAATGGCCTTGT
	LPF	TCTCCGGAATCGAACTCTAATTC
	LPB	TCTAAAGAAGAGAGGTAGTGACAAG

*LAMP* loop-mediated isothermal amplification

### Construction of positive control plasmid DNA

The sensitivity of LAMP primer assays was evaluated by using defined templates derived from plasmids constructed to include the conserved mitochondrial target (pMD19-Pan), the* 18S* sequence for *P. vivax* (pMD19-*Pv*) or the* 18S* sequence for *P. ovale* (pMD19-*Po*). The target DNA sequence was amplified with F3 and B3 LAMP primers by PCR. The amplification reactions were performed in 25 μl of PCR mixture with 1 μl DNA template, 0.5 μM of each F3 and B3 primer, 12.5 μl Master Mix (Premix Taq; Takara Taq Version 2.0 [Takara Bio Inc., Kusatsu, Japan]) and 9.5 μl DDW. The PCR cycling conditions for the amplification of the Malaria Pan mtDNA gene sequence were: 94 °C, 3 min; then 94 °C/30 s, 51.5 °C/30 s, 72 °C/1 min for 30 cycles; and a final extension at 72 °C for 5 min. The PCR cycling conditions for the amplification of the *P. vivax 18S rDNA* gene sequence were: 94 °C, 3 min; then 94 °C/30 s, 52.1 °C/30 s, 72 °C/1 min for 30 cycles; and a final extension at 72 °C for 5 min. The PCR cycling conditions for the amplification of *P. ovale 18S rDNA* gene sequence were: 94 °C for 3 min; then 94 °C/30 s, 50 °C/30 s, 72 °C/1 min for 30 cycles; and a final extension at 72 °C for 5 min. Each amplified product was sequenced and confirmed by BLAST search to correspond to the intended analyte. The target DNA fragments were then cloned into the pMD19-T Vector (Takara Bio Inc.); three recombinant plasmids were identified based on blue–white color selection.

### Limits of detection, analytical sensitivity and specificity of LAMP assays

Each recombinant plasmid was quantified using a NanoDrop ND-1000 spectrophotometer (Thermo Fisher Scientific, Waltham, MA, USA). Ten-fold serial dilutions were performed to produce plasmid concentrations as follows: pMD19-Pan from 1.08 × 10^9^ to 1.08 × 10^1^ copies/μl, pMD19-*Pv* from 1.05 × 10^9^ to 1.05 × 10^1^ copies/μl and pMD19-*Po* from 1.07 × 10^9^ to 1.07 × 10^1^ copies/μl.

Another means to compare the sensitivity of LAMP to nested PCR used a series of twofold dilutions of DNA extracted from clinical isolates from patients with *P. falciparum*, *P. vivax*, *P. ovale curtisi*, *P. ovale wallikeri* and *P. malariae* infection. The minimum concentration from which positive reactions resulted from each amplification approach was thereby determined.

The fluorescent quantitative LAMP consisted of a 12.5-μl reaction mixture with 1.6 μM FIP and BIP, 0.8 μM LpF and LpB, 0.2 μM F3 and B3, 4 mM MgSO_4_, 1.25 μL Buffer 10×, 0.5 μL *Bst* DNA polymerase (New England Biolabs), 1 μl template DNA, 0.25 μl 5× SYBR green I dye (Takara Bio Inc.) and 6 μl DDW. The reaction was performed in an isothermal device at 65 °C for 1 h using the QuantStudio 6 Flex thermocycler (Thermo Fisher Scientific). LAMP reactions were also performed as described above with Calcein-MnCl_2_ dye. PCR analyses were also performed on these positive control plasmid templates. All clinical samples and the DNA templates of *Toxoplasma gondii* were used in LAMP reactions using three sets of primers. As a control, patent LAMP primers (targeting the *18S rDNA* gene) were also employed [16]. The LAMP primers and reagents were stored separately at − 20 °C and prepared at room temperature before use.

### Statistical analysis

The clinical sensitivities, specificities and limits of detection (LODs) of *mtDNA* Malaria Pan LAMP, *18S rDNA P. vivax* LAMP and *P. ovale* LAMP were determined by considering nested PCR as the gold standard following previously reports [16, 20, 23]. Statistical comparisons were performed using SPSS version 20.0 software (SPSS IBM Corp., Armonk, NY, USA).

## Results

### Novel LAMP primers design

**Fig. 1:**
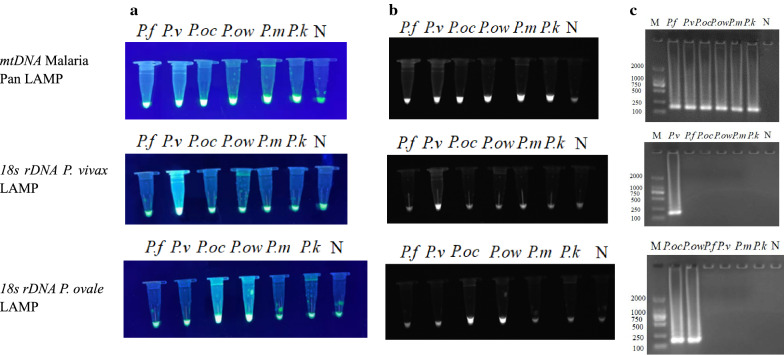
Results of *mtDNA* Malaria Pan LAMP assay, *18S rRNA Plasmodium vivax and 18S rRNA P. ovale* LAMP. **a** Observation of green fluorescence by the naked eye with UV light, **b** observation of the white fluorescence image on a Gel imager with UV light, **c** gel electrophoresis of LAMP amplifications.* LAMP* Loop-mediated isothermal amplification *P.f* *Plasmodium falciparum*, *P.v* *Plasmodium vivax*, *P.oc* *P. ovale curtisi*, *P.ow* *P. ovale wallikeri*, *P.m* *P. malariae*, *P.k* *P. knowlesi*,* M* marker,* N* negative control

Primers were designed (Table 1) that were capable of amplifying a conserved portion of the mtDNA from all five species and to design primers specific to the *18S rDNA* of either *P. vivax* or *P. ovale* (Table1). The results were observed by the naked eye with UV light, on a Gel imager or analyzed by gel electrophoresis if needed. No cross-reactions or false positives were observed (Fig. 1).

### LOD and sensitivity of LAMP primers

**Fig. 2:**
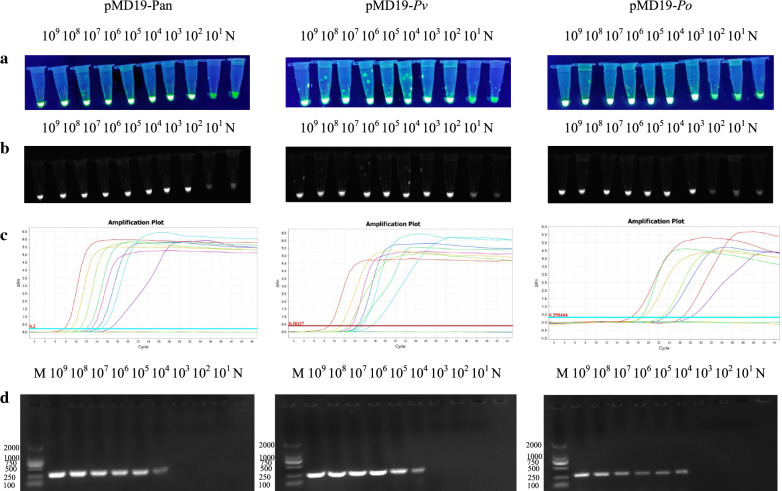
LAMP and PCR amplification of serial dilutions of pMD19-Pan, pMD19-*Pv* and pMD19-*Po*. **a** Observation of the green fluorescence by the naked eye under UV light of LAMP amplification from 10^2^ to 10^9^ copies/μl of pMD19-Pan and pMD19-*Pv* and from 10^3^ to 10^9^ copies/μl of pMD19-*Po*. **b** Observation of the white fluorescence image on the Gel imager under UV light of LAMP amplification from 10^2^ to 10^9^ copies/μl of pMD19-Pan and pMD19-*Pv* and from 10^3^ to 10^9^ copies/μl of pMD19-*Po*. **c** Amplification curves of LAMP reactions of amplification from 10^2^ to 10^9^ copies/μl of pMD19-Pan and pMD19-*Pv* and from 10^3^ to 10^9^ copies/μl of pMD19-*Po*. **d** Observation of bands following agarose gel electrophoresis (2% gel) of PCR amplification from 10^4^ to 10^9^ copies/μL of pMD19-Pan, pMD19-*Pv* and pMD19-*Po*. ΔRn value is the normalized fluorescence value obtained after deducting the baseline value.* M* Marker,* N* negative control

Using the Malaria Pan LAMP assay directed at a conserved portion of mtDNA, as few as 10^2^ copies of the positive control plasmid resulted in visibly positive reactions. By this criterion, as few as 10^2^, 10^3^ plasmid copies/μl of the *18S rDNA* of *P. vivax* and *P. ovale*, respectively, could be detected by the new LAMP assay. Analysis of amplification curves in these LAMP reactions lead to the same conclusion. By contrast, PCR assays required 10^4^ copies/μl target sequences to yield visible amplification products. By comparing the copies of plasmids/μl, the LOD of PCR was 100-fold higher than that of Malaria Pan LAMP assay and *18S rDNA P. vivax* LAMP and tenfold higher than that of *18S rDNA P. ovale* LAMP (Fig. 2).

Another means to compare the sensitivity of LAMP to PCR used a series of twofold dilutions of DNA extracted from the clinical isolates. As was true for nested PCR, each new LAMP assay was found capable of detecting < 5 parasites/μl, and no significant difference was found between the results of LAMP and nested PCR when using diluted clinical isolates. Specifically, the LOD was found to be 3.73, 3.79 and 4.15 parasites/μl for the* mtDNA* Malaria Pan LAMP assay, the *18S rDNA P. vivax* LAMP assay and the *18S rDNA P. ovale* LAMP assay, respectively. This was found to be comparable to the values obtained in the nested PCR assays (3.95, 4.58 and 4.54 parasites/μl; *P* = 0.34, 0.42 and 0.36, respectively).

### Clinical samples studied performance

A total of 279 clinical blood samples were collected from patients suspected of being infected with malaria. Parasite densities in these clinical samples, as estimated by microscopic examination, ranged from 0 to 125,240 parasite/μl blood. of these 279 blood samples, 208 were confirmed by microscopy to be malaria-positive cases and 241 were confirmed by nested PCR to be malaria-positive cases. Of the positive cases, 75 cases of *P. vivax* were in persons from Myanmar; the rest were in persons from Africa. All samples were tested by the* mtDNA* Malaria Pan LAMP assay, and the results corresponded exactly to the outcomes of the nested PCR assays (sensitivity and specificity = 100%, LOD is 3.73 ± 0.33 parasites/μL). The *18S rDNA* LAMP assay for *P. vivax* detected all 87 samples that tested positive for *P. vivax* by nested PCR, and was negative for all 192 samples judged negative by nested PCR for *P. vivax* (sensitivity and specificity = 100%, LOD is 3.79 ± 0.71 parasites/μL). The *18S rDNA* LAMP assay for *P. ovale* detected 57 of 58 isolates assessed positive by nested PCR, and assessed all 221 isolates negative that likewise tested negative by nested PCR (sensitivity = 98.28%, specificity = 100%,  LOD is 4.15 ± 0.36 parasites/μL) (Table 2)*.*

**Table 2:** Results as diagnosed by microscopic examination, nested PCR and malaria LAMP PAN assay

*Plasmodium* species	Microscopy (cases,* n*)	PCR (cases,* n*)	Malaria Pan LAMP (cases,* n*)	*P. vivax* LAMP (cases,* n*)	*P. ovale* LAMP (cases,* n*)
*Plasmodium* genus			241		
*P. falciparum*	80	92			
*P. ovale*	63	34 *P. ovale curtisi*, 24 *P. ovale wallikeri*			57
*P. vivax*	61	87		87	
*P. malariae*	4	4			
Negative	71	38	38	192	222
Total	279	279	279	279	279

Primers	Sensitivity	Specificity	LOD (parasites/μL)
Malaria Pan	100% (95% CI 98.48–100%)	100% (95% CI 90.75–100%)	3.73 ± 0.33
*P. vivax*	100% (95% CI 95.85–100%)	100% (95% CI 98.1–100%)	3.79 ± 0.71
*P. ovale*	98.28% (95%CI: 90.76–99.96%)	100% (95% CI 98.34–100%)	4.15 ± 0.36

CI, Confidence interval; LOD, limit of detection

The LAMP assays were assessed to be more sensitive than microscopy. Infection was diagnosed by microscopy in only 201 of the 241 samples deemed positive by the Malaria Pan LAMP assay (sensitivity = 81.6%); of the 38 samples deemed negative for infection by the Malaria Pan LAMP assay, microscopy identified only 31 (81.6%) as negative. Microscopy was positive for 78 of 87 clinical isolates testing positive by the LAMP assay for *P. vivax* (sensitivity = 89.7%) and microscopy was positive for 44 of 58 infections testing positive for the LAMP assay for *P. ovale* (sensitivity = 75.9%). Among the 221 samples deemed negative for *P. ovale* by the LAMP test, microscopy identified 201 (91%) as negative for *P. ovale.* Overall, the LAMP tests provided greater correspondence to results from nested PCR and were deemed of greater diagnostic value than microscopy (*P* < 0.05).

Previously described LAMP primers [14] were applied to 30 randomly selected clinical samples, yielding a sensitivity of 93% (95% confidence interval [CI] 74.97–99.02%) and a specificity of 80% (95% CI 78.88–99.89%). Among 25 positive samples, one sample infected with *P. falciparum* and one sample infected with *P. ovale wallikeri* tested negative with the previously reported LAMP assay. This assay assessed four of the five negative samples as uninfected (Table 3)*.* Thus, these primers afforded lower sensitivity and specificity than the newly described LAMP assay.

**Table 3:** Correspondence of three assays for detecting *Plasmodium* infections

*Plasmodium* genus	Number of cases		
	Nested PCR	*mtDNA* Malaria Pan LAMP	Malaria genus-specific LAMP patent primer (Japan)
Positive	25	25	24
Negative	5	5	6
Total	30	30	30
*P* value		< 0.01	< 0.01

## Discussion

The World Health Organization has targeted malaria for eradication by 2030. The prevalence of malaria has been declining over the long term, but no significant progress in case reduction has been accomplished in the past 4 years [24] and 57 persons per 1000 general population were still at risk of infection in 2018 [1]. Rapid diagnosis plays an important role in pursuing this goal of total malaria eradication. The traditional microscopic examination method is still widely used in many malaria-endemic areas due to limited public health/medical facilities and lower economic levels; in addition, technical proficiency is required. In our study, microscopy was found to be less sensitive and less specific than the newly described LAMP tests. Technical experience of local operators and existence of submicroscopic malaria greatly impacted the accuracy of detection. LAMP and PCR identified infection in some clinical samples in which no blood-stage parasites were detected by microscopy.

LAMP, as a new and easy-to-operate molecular diagnostic method, has the potential to be broadly applied and is considered to have the possibility of replacing the classic PCR method. Our specific aim was to detect, with requisite sensitivity and specificity, those infections capable of persisting as hypnozoites. The elimination of these liver stages of *P. vivax* and *P. ovale* is important to preclude clinical relapse. The target gene sequence *mtDNA* of the Malaria Pan LAMP assay occurs as 30–150 copies per parasite; the *18S rDNA* of *P. vivax* and *P. ovale* is present as four to eight copies per parasite. Intraspecific sequence conservation has been confirmed for each species [22, 24, 25].

A previous report [20] warned of cross-reactions ensuing from the LAMP primers described by Han et al. [16]. We found that these genus-wide LAMP primers had a lower sensitivity and specificity. A set of Malaria Pan LAMP primer targeting the *18S rDNA* sequence carried out in Malaysia had comparable sensitivity and specificity to our assay [23]. The reported LOD was lower than that of our assay and values reported previously; indeed, a LOD of only one copy raises concerns of reproducibility [26, 27].

The commercially available Eiken Loopamp™ MALARIA Pan Detection Kit targeting the *18S rDNA* sequence with 83.4% sensitivity and 81.58% specificity was found not to be suitable for clinical diagnosis; in particular, its sensitivity was only 50% when parasitemia was < 50 parasites/μl blood [20]. Although the commercially available Illumigene Malaria Plus test targeting mtDNA sequence showed good sensitivity and specificity of > 98%, respectively [28], it has been reported to have false negative tests in cases where parasitemia with *P. falciparum* exceeded 1% [20].

The performance of our *18S rDNA P. vivax* LAMP with 100% sensitivity and 100% specificity and *18S rDNA P. ovale* LAMP with 100% sensitivity and 100% specificity was also similar or better than previous reported species-specific LAMP assays [20, 24, 26]. The LOD of our Malaria Pan LAMP, *P. vivax* LAMP and *P. ovale* LAMP was 3–4 parasites/μl, which is close to the LOD of nested PCR; previous reports have found comparable sensitivities of other LAMP assays [29]. Compared to the LOD of the PCR assay (10^3^ copies/μl), the LODs of our Malaria Pan LAMP (10^2^ copies/μl) and *P. vivax* LAMP (10^2^ copies/μl) were 100-fold lower, and of our *P. ovale* LAMP, tenfold lower (10^4^ copies/μL). These results are similar to those reported previously for other LAMP assays [26, 27].

The primers used in the present study differ from previous assays (Additional file 1: Table S1) and have shown high sensitivity and specificity. For the first time, LAMP was evaluated for its diagnostic performance of *P. ovale* in a large-scale study (279 clinical samples). Reported infections with *P. ovale* and *P. vivax* have increased among Chinese migrant laborers in Africa [30–39]. The performance and convenience of the novel LAMP assays described here offer promise as tools for clinical diagnosis and epidemiological surveys. In cases where parasites in the genus *Plasmodium* have first been identified, differential diagnosis can then be accomplished using species-specific assays for *P. vivax* and *P. ovale*.

## Conclusions

We have described three sets of novel LAMP assays for the detection of malaria. The first, targeting a conserved portion of the *mtDNA* gene, detects all five malaria species known to infect people. The other two, targeting portions of the *18S rDNA* gene, specifically amplify *P. vivax* or *P. ovale*, the two species prone to persisting as hypnozoites and causing clinical relapse if not recognized and eliminated through chemotherapies directed at those liver stages.

We have shown that these assays are highly sensitive and specific when applied to defined control templates and clinical isolates. The assays are easy to perform and require no complicated experimental equipment. This diagnostic approach can assist a wide range of research and clinical applications.

## Supplementary Information

**Additional file 1: Table S1.** *Plasmodium falciparum*, *P. vivax*, *P. ovale curtisi*, *P.ovale wallikeri*, *P. malariae and P. knowlesi* *mtDNA* sequences, *P. vivax 18S rRNA* sequences and *P. ovale curtisi*, *P. ovale wallikeri 18S rDNA* sequences.

## Data Availability

All data generated or analyzed during this study are included in this published article and its supplementary information file. The datasets of clinical malaria samples used and analyzed during the present study are available from the corresponding author on reasonable request.

## Abbreviations

LAMP: Loop-mediated isothermal amplification
LOD: Limit of detection
*mtDNA*: Mitochondrial DNA gene
*18S rDNA*: Nuclear 18S ribosomal RNA gene
