# Supplementary material for: Loop-mediated isothermal amplification (LAMP) assays targeting 18S ribosomal RNA genes for identifying P. vivax and P. ovale species and mitochondrial DNA for detecting the genus Plasmodium
Source: Parasit Vectors. 2021 May 24;14:278. doi: 10.1186/s13071-021-04764-9 (PMC8147439; doi:10.1186/s13071-021-04764-9)
Supplement: Supplementary file 1 — Additional file 1: Table S1. Plasmodium falciparum, P. vivax, P. ovale curtisi, P.ovale wallikeri, P. malariae and P. knowlesi mtDNA sequences, P. vivax 18S rRNA sequences and P. ovale curtisi, P. ovale wallikeri 18S rDNA sequences. [file 13071_2021_4764_MOESM1_ESM.doc]

Table S1. *P. falciparum*, *P. vivax*, *P. ovale curtisi*, *P.ovale wallikeri*, *P. malariae* and *P. knowlesi* *mtDNA* sequences, *P. vivax 18s rDNA* sequences and *P. ovale curtisi*, *P.ovale wallikeri 18s rDNA* sequences.

| *Plasmodium* species and subspecies | Gene name | Sequences (5’-3’) |
| --- | --- | --- |
| *P. falciparum* | *mtDNA* | GGTTCAGCTACAAGTTCACTGTCAACTACCATGTTACGACTTCGCACCGACTGTTT-CTTTTACCTCACGAGTCGATCAGGAAGGTTTCATCCTTAAATCTCGTAACCATGCCAACACATAAGAACTTTT-AGGGAAGTTAAGGTGCTCAGGGTCTTACCGTCGGGCCGTATGATTCCACATATTCATGGATAATTCTATTTATTAGGAGTCTCACACTAGCGACAATGGGGAAGTCGTTACACCGTTCATGCAGGACGGAGATTACCCGACAAGGAATTTTGCTACCTTAGGACCGTTTA |
| *P. vivax* |  | GGTTCAGCTACAAGTTCACTGTCAACTACCATGTTACGACTTCGCACCGACTGTTTTCTTTTACCTCACGAGTCGATCAGGAAGGTTTCATCCTTAAATCTCGTAACCATGCCAACACATAAGAACTTTTTAGGGAAGTTAAGGTGCTCAGGGTCTTACCGTCGGGCCGTATGATTCCACATATTCATGGATAATTCTATTTATTAGGAGTCTCACACTAGCGACAATGGGGAAGTCGTTACACCGTTCATGCAGGACGGAGATTACCCGACAAGGAATTTTGCTACCTTAGGACCGTTTA |
| *P. ovale curtisi* |  | GGTTCAGCTACAAGTTCACTGTCAACTACCATGTTACGACTTCGCACCGACTGTTTTCTTTTACCTCACGAGTCGATCAGGAAGGTTTCATCCTTAAATCTCGTAACCATGCCAACACATAAGAACTTTTTAGGGAAGTTAAGGTGCTCAGGGTCTTACCGTCGGGCCGTATGATTCCACATATTCATGGATAATTCTATTTATTAGGAGTCTCACACTAGCGACAATGGGGAAGTCGTTACACCGTTCATGCAGGACGGAGATTACCCGACAAGGAATTTTGCTACCTTAGGACCGTTTA |
| *P.ovale wallikeri* |  | GGTTCAGCTACAAGTTCACTGTCAACTACCATGTTACGACTTCGCACCGACTGTTT-CTTTTACCTCACGAGTCGATCAGGAAGGTTTCATCCTTAAATCTCGTAACCATGCCAACACATAAGAACTTTTTAGGGAAGTTAAGGTGCTCAGGGTCTTACCGTCGGGCCGTATGATTCCACATATTCATGGATAATTCTATTTATTAGGAGTCTCACACTAGCGACAATGGGGAAGTCGTTACACCGTTCATGCAGGACGGAGATTACCCGACAAGGAATTTTGCTACCTTAGGACCGTTTA |
| *P. malariae* |  | GGTTCAGCTACAAGTTCACTGTCAACTACCATGTTACGACTTCGCACCGACTGTTT-CTTTTACCTCACGAGTCGATCAGGAAGGTTTAATCCTTAAATCTCGTAACCATGCCAACACATAAGAACTTTT-AGGGAAGTTAAGGTGCTCAGGGTCTTACCGTCGGGCCGTATTATTCCACATATTCATGGATAATTCTATTTATTAGGAGTCTCACACTAGCGACAATGGGGAAGTCGTTACACCGTTCATGCAGGACGGAGATTACCCGACAAGGAATTTTGCTACCTTAGGACCGTTTA |
| *P. knowlesi* |  | GGTTCAGCTACAAGTTCACTGTCAACTACCATGTTACGACTTCGCACCGACTGTTTTCTTTTACCTTACGAGTCGATCAGGAAGGTTTCATCCTTAAATCTCGTAACCATGCCAACACATAAGAACTTTTTAGGGAAGTTAAGGTGCTCAGGGTCTTACCGTCGGGCCGTATGATTCCACATATTCATGGATAATTCTATTTATTAGGAGTCTCACACTAGCGACAATGGGGAAGTCGTTACACCGTTCATGCAGGACGGAGATTACCCGACAAGGAATTTTGCTACCTTAGGACCGTTTA |
| *P. vivax* | *18s rDNA* | ACGAACGAGATCTTAACCTGCTAATTAGCGGTAAGTACGACATATTTTTATGTCGGATTGGATCTGGATGATTTGCTTATATTGAGGTGCAATCTAAATAGGGGATTGCAATTATACTTCGTGTCGGTGTTTCTTAATCGAATAGCTGATGCGTTTGGTATATTGCTTTCCTTTTTTTTTATTTCTGCGCTTCTTTACTTGGCTTATCGTACCGTTTCCTTTTTGTGTAGAAATGTATTTGCATTATATTAAAGCTTCTTAGAGGAACGATGTGTGTCTAACACAAGGAAGTTTAAGGCAACAACAGGTCTGTGATGTCCTTAGATGAACTAGGCTGCACGCGTGCTACACTGATATGTACAACGAGTT |
| *P. ovale curtisi* | *18s rDNA* | CCTTATTTAGTGTGTATCAATCGAGTTTCTGACCTATCAGCTTTTGATGTTAGGGTATTGGCCTAACATGGCTATGACGGGTAACGGGGAATTAGAGTTCGATTCCGGAGAGGGAGCCTGAGAAATAGCTACCACATCTAAGGAAGGCAGCAGGCGCGTAAATTACCCAATTCTAAAGAAGAGAGGTAGTGACAAGAAATAACAATACAAGGCCATTTCATGGTTTTGTAATTGGAATGATGGGAATTTAAAACCTTCCCAAAATTCAATTGGAGGGCAAGTCTGGTGCCAGCAGCCGCGGTAATTCCAGCTCCAATAGCGTATATTAAAATTGTTGCAGTTAAAACGCTCGTAGTTGAATTTCAAAGAATCAATATTTTAAGTAAT |
| *P.ovale wallikeri* |  | CCTTATTTAGTGTGTATCAATCGAGTTTCTGACCTATCAGCTTTTGATGTTAGGGTATTGGCCTAACATGGCTATGACGGGTAACGGGGAATTAGAGTTCGATTCCGGAGAGGGAGCCTGAGAAATAGCTACCACATCTAAGGAAGGCAGCAGGCGCGTAAATTACCCAATTCTAAAGAAGAGAGGTAGTGACAAGAAATAACAATACAAGGCCATTTCATGGTTTTGTAATTGGAATGATGGGAATTTAAAACCTTCCCAAAATTCAATTGGAGGGCAAGTCTGGTGCCAGCAGCCGCGGTAATTCCAGCTCCAATAGCGTATATTAAAATTGTTGCAGTTAAAACGCTCGTAGTTGAATTTCAAAGAATCAATATTTTAAGTAAT |
